# Effect of the Peiyu granules on early miscarriage among women undergoing embryo transfer: a randomized, double-blind, placebo-controlled trial

**DOI:** 10.3389/fendo.2025.1631313

**Published:** 2025-09-09

**Authors:** Dandan He, Tianyi Lyu, Xiaonan Wang, Yanmin Ma, Yonglian Lan, Xiaokui Yang, Chanwei Jia, Liying Zhou, Yu Liang, Ying Li, Yinmei Dai, Wentao Yue, Ruixia Liu, Zhen Liu, Chen Ma, Dan Liu, Ying Wu, Chenghong Yin

**Affiliations:** ^1^ Department of Acupuncture, Beijing Obstetrics and Gynecology Hospital, Capital Medical University, Beijing Maternal and Child Health Care Hospital, Beijing, China; ^2^ Department of Epidemiology and Health Statistics, School of Public Health, Capital Medical University, Beijing, China; ^3^ Department of Reproductive Medicine, Beijing Obstetrics and Gynecology Hospital, Capital Medical University, Beijing Maternal and Child Health Care Hospital, Beijing, China; ^4^ Department of Good Clinical Practice, Beijing Obstetrics and Gynecology Hospital, Capital Medical University, Beijing Maternal and Child Health Care Hospital, Beijing, China; ^5^ Department of Central Laboratory, Beijing Obstetrics and Gynecology Hospital, Capital Medical University, Beijing Maternal and Child Health Care Hospital, Beijing, China

**Keywords:** Peiyu granules, early miscarriage, embryo transfer, RCT - randomized controlled trial, *in vitro* fertilization (IVF)

## Abstract

**Objective:**

To evaluate the efficacy and safety of the Peiyu Granules (PYG) compared with placebo on early miscarriage rates among women undergoing embryo transfer.

**Methods:**

A double-blind, parallel-group randomized clinical trial between February 15, 2017, and June 17, 2019, within 10 months of pregnancy follow-up until March 2020. This clinical trial was conducted at Beijing Obstetrics and Gynecology Hospital, Capital Medical University. A total of 886 women were included in this study. The intervention group (n = 443) received PYG on the night of Embryo Transfer (ET) until the day of the hCG test. If it was negative, the patient stopped taking medicine. In contrast, the treatment continued until 70 days after ET. The women in the control group (n = 443) consumed the same amount of placebo as the intervention group. All women enrolled were subject to the same follow-up protocols. The primary outcome was early miscarriage rate. The secondary outcomes were clinical intrauterine pregnancy rate and live birth rate.

**Results:**

Among the 886 randomized women (mean [SD] age, 32.8 [3.6] years), 854 women (96.4%) underwent ET and followed the treatment of random grouping. Early miscarriage occurred among 17 of 133 women (12.8%) receiving PYG compared with 35 of 156 women (22.4%) receiving placebo (relative risk[RR], 0.51 [95% CI, 0.27 to 0.95], P = 0.02). Clinical intrauterine pregnancy rates were 30.0% (133 of 443) in the intervention group and 35.2% (156 of 443) in the control group (relative risk[RR], 0.79 [95% CI, 0.60 to 1.05], P = 0.10). Live-birth rates were 25.3% (112 of 443) in the intervention group and 25.7% (114 of 443) in the control group (relative risk[RR], 0.98 [95% CI, 0.72 to 1.32], P = 0.88). Live birth rates in the clinical pregnant population were 84.2% (112/133) in the intervention group and 73.7% (115/156) in the control group (relative risk [RR], 1.14 [95% CI, 1.01 to 1.29], P = 0.03).

**Conclusion:**

The findings suggested that PYG reduced early miscarriage rates among women undergoing embryo transfer. However, there were no significant improvement in clinical pregnancy rates and live birth rates.

**Clinical trial registration:**

https://www.chictr.org.cn/showproj.html?proj=12343 identifier, ChiCTR-inr-16010087.

## Introduction

Infertility is a serious disease (1, 2) and a public health problem (3) that is increasingly recognized and considered. Assisted reproductive technology (ART), as one of the important methods to treat infertility, has unsatisfactory success rates, still hovering between 30%-40% (4–6). At present, it is difficult to make a breakthrough in improving the pregnancy outcome of ART with Western medicine alone, so an increasing number of researchers are gradually concerned with traditional Chinese medicine, which is considered a complementary or alternative medicine for infertility treatment (7–10).

Growing evidence has revealed that Chinese herbal medicines (CHMs) can improve pregnancy outcomes among infertile couples undergoing ART treatment (8, 11–14). However, most of them focus on using CHMs before or during *in vitro* fertilization (IVF) to optimize egg quality (15, 16), ovarian function or endometrial receptivity (17, 18). Until we began this study, only a few studies had focused on the use of CHMs after embryo transfer to reduce the miscarriage rate (13). Infertile couples have a higher frequency of spontaneous abortions than the general population (19). Besides, spontaneous abortion rate of frozen thawed embryo transfer cycle is also high (20, 21). With the implementation of China’s two-child and three-child policies, the age of infertile women in demand for ART is increasing (22, 23). However, early miscarriage rates are high and fertility rates are low in old age group (24, 25). Meanwihle, a high early miscarriage rate seriously threatens the live birth rate of ART. For thousands of years, CHMs used to prevent and treat miscarriage have been popular in Asian countries (26, 27).

Peiyu Granules are a TCM compound granule preparation composed of 19 herbals (**Supplementary Table S1**). It was created by Professor Zhao Quansong, a highly regarded doctor nationwide. Moreover, PYG has showed great clinical efficacy and safety to prevent and treat miscarriage for decades. Previous clinical trial has demonstrated that PYG significantly upregulates luteinizing hormone and progesterone in patients with threatened miscarriage (28). Further foundational study has revealed that PYG enhances invasion and proliferation capability of human trophoblast cell (29). Grounded on initial clinical findings and experimental evidence, we hypothesize that PYG could decrease the early miscarriage rates among women undergoing embryo transfer, and conducted a large-scale, double-blind, randomized, placebo-controlled trial to evaluate its efficacy and safety.

## Methods

### Study design

This single-center, randomized, double-blind, placebo-controlled superiority trial was conducted in Beijing Obstetrics and Gynecology Hospital, Capital Medical University. This trial followed the Consolidated Standards of Reporting Trials (CONSORT) Extension for Chinese Herbal Medicine Formulas 2017 reporting guideline.

### Ethical approval

The trial was approved by the ethics committee of Beijing Obstetrics and Gynecology Hospital (IEC-C-29-V01.1) under the ethics approval number 2016-KY-082-01. The study protocol was approved by all 12 review committee members who attended the meeting and voted, including 12 independent members and 2 with TCM-related professional backgrounds. All eligible women signed written informed consent.

### Participants

We recruited participants who intended to undergo ET from the reproductive center of Beijing Obstetrics and Gynecology Hospital, Capital Medical University. Participants were enrolled from February 15, 2017 to June 17, 2019. Follow-up of live births were completed in April 2020.

The inclusion criteria were 1) women with infertility aged between 22 and 40, and 2) body mass index (calculated as weight in kilograms divided by height in meters squared) of 35 or less.

The exclusion criteria were 1) Women with three or more spontaneous miscarriages; 2) severe endometriosis, including adenomyosis of the uterus and ovarian “chocolate” cysts; 3) anatomical cacogenesis of uterus (eg, uterus unicornis, arcuate uterus, septate uterus, etc); 4) untreated bilateral hydrosalpinx; 5) untreated endometrial diseases (eg, endometritis, endometrial polyp); or 6) diseases unsuitable for ART or pregnancy.

### Randomization and masking

The block randomization method was used. The length of the block was 4 and SAS9.4 (SAS Institute Inc., Cary, NC, United States) was used to generate a randomization sequence for 884 subjects (intervention group, control group) according to a 1:1 ratio and list the treatment allocation corresponding to serial numbers 001–884 (that is, a random coding table). The last two patients were assigned to intervention group, control group using a simple randomization method, with the randomization sequence generated by SAS software. After being introduced to the trial procedure, eligible women were randomly divided into either the intervention group or the placebo control group. The randomization sequence was independently generated by a statistician using SAS. To ensure allocation concealment, the subject number, random number and corresponding drug number were obtained only by inputting the eligible women’s birth date into the Central Randomization System for Clinical Research (Web Edition). Investigators, women enrolled, clinical trial coordinators and analysts were blinded to group allocation. The allocation was individually sealed by a statistician in an opaque envelope. Blinding was maintained until statistical analysis was completed. In the event of a medical emergency, unblinding was permitted. The reason and time for unblinding was well documented and initialed by the supervising physician.

### Intervention

Women were recruited on the day of ET and randomly assigned in a 1:1 ratio to receive traditional Chinese medicine PYG [a dose of 3 bags (10.1 g/bag), 2 times a day] or matching placebo [a dose of 3 bags (10.1 g/bag), 2 times a day]. PYG and placebo were provided by Jiangyin Tianjiang Medicine Co. Ltd. (China), and their preparations are provided in **Supplementary Table S1**. The packaging and granules of placebo and PYG are identical in appearance, smelling and taste. Following randomization, women with special prescription for this study were taken to Good Clinical Practice (GCP) pharmacy for PYG or placebo by clinical trial coordinator. The two persons in charge gave the drugs and diary cards to women after checking their basic information and prescription, concurrently informing them of the drugs taking method, matters needing attention and next follow-up time.

The first treatment course was from the night of ET to the day of the hCG test (generally 12–14 days after ET). If it was positive (hCG > 10 miu/ml), the second treatment course started and remained the same until the day of transvaginal ultrasound. Medication was continued until the 70th day after ET if women achieved clinical pregnancy. When a woman was not pregnant after ET, drug treatment and follow-up were terminated. Adherence to medication was monitored by requiring women to return any unused drugs, packages of the drugs and diary cards that recorded the details of daily medications. A trained researcher recorded pregnancy outcomes at the 12th week of gestational age and after birth by follow-up telephone.

Eligible women in both groups received the same western medicine luteal support protocol. Oral progesterone capsules (Zhejiang Xianju Pharmaceutical Co. Ltd., Chinese medicine approval: H20041902, 100 mg each time, twice a day) combined with vaginal progesterone soft capsules (Besins Healthcare Benelux, Imported Drug Registration No. H20160265, Spain, 0.2 g each time, three times a day) from the day of ET until the 10th week of pregnancy.

### Sample size calculation

A survey showed that the risk of pregnancy loss among women under 33 years using their own oocytes and freshly fertilized embryos was 22% (30). Combined with the pre-experimental results, it was assumed that Peiyu Granules intervention could reduce the risk of pregnancy loss by half. Pregnancy loss rate decreased from 22% to 11%, requiring 175 clinical pregnancies per group (α error, 0.05; β error,0.2). The clinical pregnancy rate at the Center of Reproductive Medicine of Beijing Obstetrics and Gynecology Hospital, Capital Medical University, was 41.5%. And assuming a 5% dropout rate, the total number of women was 443 for each group.

### Outcomes

The primary outcome was early miscarriage, defined as the loss of a viable intrauterine pregnancy up to and including gestational week 12 + 0. Secondary outcomes included clinical intrauterine pregnancy, late miscarriage and live births. Ultrasonography showing an intrauterine sac with or without a fetal heart could be diagnosed as clinical intrauterine pregnancy (31). Late miscarriage was defined as pregnancy loss between 13 and 28 weeks of gestation (32). Live births were defined as the delivery of at least one living infant (33). In addition, women with persistent pregnancy were followed up until delivery. The delivery mode, gestational age and birth weight were all recorded. The adverse events of the intervention were detailed from the beginning to the end of the study.

### Statistical analysis

Double-entry is applied by using the ClinResearch Electronic Data Capture (V4.0). Analyses are performed after the data was checked by inspectors. The main outcomes are compared between two groups using chi-square test and risk differences and relative risks with associated 95% Confidence Intervals. Comparison between groups was performed with independent sample t test or Mann-Whitney test, or Pearson chi-square test or fisher’s exact test as appropriate.

The intention-to-treat (ITT) and per-protocol methods were used to analyze primary and secondary end points, as well as safety indexes. ITT analysis was defined as participants who were randomly allocated to intervention and control group, regardless of whether they actually received the treatment or not. Participants who were lost to follow up were counted as no clinical pregnancy in intention-to-treat analysis. Best-case and worst-case sensitivity analysis were conducted on early miscarriage, clinical intrauterine pregnancies and live birth if participants were lost to follow-up. In best-case analysis, unknown events were defined as most favorable assumptions for PYG and least favorable assumptions for placebo. In worst-case analysis, unknown events were defined as least favorable assumptions for PYG and most favorable assumptions for placebo. All Statistical analyses were conducted using the statistical package SPSS, version 26.0 (IBM Corp). Statistical significance was defined as *P* < 0.05 with two tails.

## Results

### Enrollment and baseline characteristics of participants

**Figure 1:**
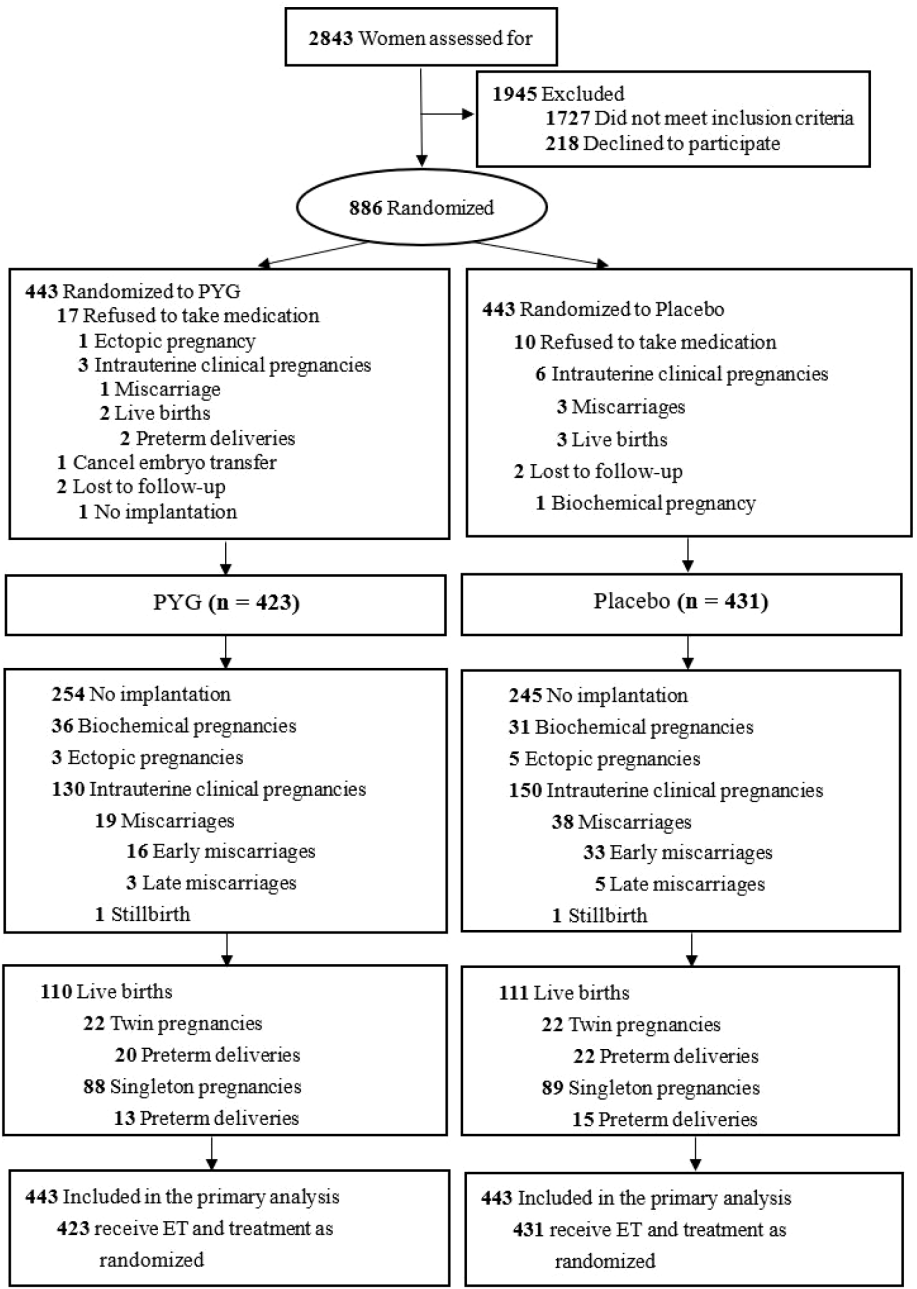
Participant flow chart. PYG, Peiyu Granule; ET, embryo transfer.

Between February 15, 2017, and June 17, 2019, a total 886 of 2843 eligible women were enrolled and randomly divided into intervention group (n=443) and control group (n=433). The flowchart was showed in **Figure 1**. After randomization, 32 participants withdrew (20 in intervention group and 12 in control group). Among the 32 withdrawers, 27 participants refused to take medication (17 in intervention group and 10 in control group). 4 participants lost to follow up (2 in intervention group and 2 in control group). 1 participant in intervention group canceled embryo transfer. We collected efficacy and safety outcomes of drop-out participants through medical record reviews or telephone follow-ups. 2 participants (1 in intervention group and 1 in control group) lost to follow-up. Participants who lost to follow up were counted as no clinical pregnancy in intention-to-treat analysis. Finally, 886 participants (433 in intervention group and 433 in control group) comprised the ITT analysis for subsequent analysis (per-protocol set, **Supplementary Table S2**).

As presented in **Table 1**, intervention group and control group have similiar baseline characteristics in age, BMI, primary infertility, duration of infertility, previous spontaneous miscarriages, occupation, education background, causes of infertility, follicle-stimulating hormone, luteinizing hormone, estradiol, testosterone, prolactin, progesterone and thyroid-stimulating hormone.

**Table 1:** Baseline characteristics of the participants in intervention and control groups (intention-to-treat analysis).

Characteristic	Intervention	Control	*P* value
	(n = 443)	(n = 443)	
Age, mean (SD), y	32.8 (3.7)	32.8 (3.6)	.791
Body mass index, mean (SD)^a^	23.2 (3.8)	23.3 (3.9)	.669
Primary infertility, No. (%)	219 (49.9)	243 (55.0)	.289
Duration of infertility, median (IQR), y	3 (2-5)	4 (2-5)	.258
Previous spontaneous miscarriages, No. (%)			.156
0	234 (52.8)	265 (59.8)	
1	133 (30.0)	106 (23.9)	
2	66 (14.9)	64 (14.4)	
3	10 (2.3)	8 (1.8)	
Occupation, No. (%)			.665
Unemployed	130 (29.3)	128 (28.9)	
Blue-collar worker	41 (9.3)	34 (7.7)	
White-collar worker	272 (61.4)	281 (63.4)	
Education background, No. (%)			.883
Junior high school and below	43 (9.7)	44 (9.9)	
Senior high school	95 (21.4)	89 (20.1)	
University degree or above	305 (68.8)	310 (70.0)	.371
Causes of infertility, No. (%)			
Male factors	168 (37.92)	182 (41.08)	
Female factors			
Tubal factor	347 (78.3)	322 (74.9)	.132
Endometriosis	26 (5.87)	33 (7.45)	.419
Diminished ovarian reserve	46 (10.38)	38 (8.58)	.422
Polycystic ovary syndrome	60 (13.54)	72 (16.25)	.299
Intrauterine insemination failure	80 (18.06)	83 (18.74)	.862
Unknown factors	20 (4.51)	23 (5.19)	.755
Follicle-stimulating hormone, median (IQR), IU/L	7.02 (5.97-8.01)	6.96 (5.87-8.18)	.967
Luteinizing hormone, median (IQR), IU/L	3.80 (2.83-5.25)	3.87 (2.79-5.29)	.885
Estradiol, median (IQR), pg/mL	43.36 (35.53-51.82)	42.86 (34.26-53.43)	.738
Testosterone, median (IQR), ng/dL	36.86 (28.00-46.34)	36.29 (28.86-46.00)	.938
Prolactin, median (IQR), ng/mL	11.03 (8.28-15.31)	11.55 (8.61-16.53)	.283
Progesterone, median (IQR), ng/mL	0.54 (0.38-0.72)	0.51 (0.37-0.71)	.401
Thyroid-stimulating hormone, median (IQR), mIU/L	1.90 (1.43-2.58)	1.95 (1.44-2.69)	.338

SD, standard deviation; IQR, interquartile range.

a Body mass index is the weight in kilograms divided by the square of the height in meters.

### Protocols and IVF-ET data in participants

The percentage of fresh and frozen thawed embryo transfer, distribution of protocols for controlled ovarian hyperstimulation, times of ET, stage of embryos transferred and number of transferred embryos did not significantly differ between groups (**Table 2**, **Supplementary Table S3**).

**Table 2:** Protocols of controlled ovarian hyperstimulation and data of *in vitro* fertilization and embryo transfer (intention-to-treat analysis).

Characteristic	No. (%) of participants		*P* value
	Intervention (n = 443)	Control (n = 443)	
Types of embryo transfer			
Fresh embryo transfer	159 (36.0)	168 (38.0)	.54
Frozen thawed embryo transfer	284 (64.0)	275 (62.0)	
Hormone replacement of frozen thawed embryo transfer	173 (60.5)	175 (63.5)	.47
Natural cycle of frozen thawed embryo transfer	111 (39.5)	100 (36.5)	
Protocol of controlled ovarian hyperstimulation			
Ultralong GnRH agonist	42 (9.6)	52 (11.5)	.73
Long GnRH agonist	138 (31.4)	128 (29.0)	
Short GnRH agonist	56 (12.5)	55 (12.4)	
GnRH antagonist	207 (46.5)	208 (47.1)	
Times of ET			
0	199 (44.9)	209 (47.2)	.57
1	131 (29.6)	120 (27.1)	
2	62 (14.0)	71 (16.0)	
3 or more	51 (11.5)	43 (9.7)	
Number of embryos transferred			
1	93 (21.0)	87 (19.6)	.58^a^
2	348 (78.6)	353 (79.7)	
3	2 (0.5)	3 (0.7)	
Stage of embryos transferred			
Blastocyst stage	360 (81.1)	344 (77.6)	.20
Cleavage stage	83 (18.9)	99 (22.4)	

GnRH, gonadotropin-releasing hormone; ET, embryo transfer.

a Mann-Whitney U Test.

### Primary outcome

There was significant difference in the early miscarriage between intervention group and control group. As shown in **Table 3**, the early miscarriage rate was 12.8% (17/133) in intervention group compared with 22.4% (35/156) in the control group according to the intention-to-treat analysis (rate ratio[RR], 0.51[95%CI,0.27 to 0.95]; *P*=0.03).

**Table 3:** Pregnancy outcomes of the participants (intention-to-treat analysis).

IVF-ET outcomes	No./Total no. (%)		Relative risk^d^ (RR), (95% CI)	*P* value
	Intervention	Control		
Primary outcome				
Early miscarriages	17/133 (12.8)	35/156 (22.4)	0.51 (0.27 to 0.95)	.03
Secondary Outcome				
Miscarriages^a^	20/133 (15.0)	41/156 (26.3)	0.50 (0.27 to 0.90)	.02
Late miscarriages	3/133 (2.3)	6/156 (3.8)	0.58 (0.14 to 2.35)	.66^e^
Clinical intrauterine pregnancies	133/443 (30.0)	156/443 (35.2)	0.79 (0.60 to 1.05)	.10
Twin pregnancies^b^	27/133 (20.3)	32/156 (20.5)	0.99 (0.56 to 1.75)	.96
Live births	112/443 (25.3)	114/443 (25.7)	0.98 (0.72 to 1.32)	.88
Live births among clincal pregnancies	112/133 (84.21)	115/156 (73.72)	1.14 (1.01 to 1.29)	.03
Preterm deliveries^c^	35/112 (31.3)	37/114 (32.5)	0.95 (0.54 to 1.66)	.85
Birth weight (g)				
Singleton	3,350 (3,055 - 3,575)	3,420 (3,150 - 3,763)	NA	.26
Twin	2,648 (2,311 - 2,910)	2,515 (2,275 - 2,699)	NA	.14
Gestational age (weeks)				
Double live fetus	35.8 ± 2.2	36.1 ± 1.3	NA	.66
Single live fetus	38.6 ± 2.1	38.6 ± 1.7	NA	.92
Delivery mode				
Twin births				
A vaginal delivery	2/23 (8.7)	2/22 (9.1)	0.96 (0.15-6.21)	1^e^
Cesarean section	21/23 (91.3)	20/22 (90.9)	1.00 (0.84-1.21)	
Single births				
A vaginal delivery	40/89 (44.9)	41/93 (44.1)	1.02 (0.74-1.41)	.907
Cesarean section	49/89 (55.1)	52/93 (55.9)	0.98 (0.76-1.28)	
Adverse event	71/443 (16.0)	93/443 (21.0)	0.76 (0.58 to 1.01)	.06

^a^Miscarriage was defined as pregnancy loss before the 28th week of gestation; the miscarriage rate was defined as miscarriages per clinical intrauterine pregnancy.

^b^Twin pregnancy rate was defined as twin pregnancies per clinical intrauterine pregnancies.

^c^Premature delivery was defined as a live birth before 37 weeks of gestation; Preterm delivery rate was defined as preterm deliveries per live births.

^d^95% CIs of ARD were calculated using VassarStats.

^e^Yates’s correction for continuity.

### Secondary outcomes

As shown in **Table 3**, there was no significant differece in late miscarriage, clinical intrauterine pregnancy, and live births. The clinical intrauterine pregnancies rate is 30% (133/443) in the intervention group vs 35.2% (156/443) in the control group (rate ratio[RR], 0.79[95%CI,0.60 to 1.05]; *P*=0.10). The late miscarriage rate is 2.3% (3/133) in the intervention group vs 3.8% (6/156) in the control group (rate ratio[RR], 0.58[95%CI,0.14 to 2.35]; *P*=0.51). The total live births rate is 25.3% (112/433) in the intervention group vs 25.7% (114/443) in the control group (rate ratio[RR], 0.98[95%CI,0.72 to 1.32]; *P*=0.88). Live birth rates in the clinical pregnant population were 84.2% (112/133) in the intervention group and 73.7% (115/156) in the control group (relative risk[RR], 1.14 [95% CI, 1.01 to 1.29], P = 0.03). As shown in **Table 3** and **Supplementary Table S7**, a total of 164 women suffered from adverse events, with no significant difference between groups (16.0% vs 21.0%; rate ratio[RR], 0.76 [95% CI, 0.58 to 1.01]).

### *Post hoc* outcomes

The *post hoc* per-protocol analysis remained consistent with the intention-to-treat analysis (**Supplementary Table S4**). Meanwhile, not only the best-case sensitivity analysis but also the worst-case sensitivity analysis exhibited uniformity in both intention-to-treat and per-protocol sets (**Table 4**, **Supplementary Table S5**).

**Table 4:** *Post hoc* sensitivity analysis for pregnancy outcomes of the participants (intention-to-treat analysis).

IVF-ET outcomes	No./Total no. (%)		Relative risk^d^ (RR), (95% CI)	*P* value
	Intervention	Control		
Best case for PYG^a^				
Early miscarriages^b^	17/134 (12.7)	35/157 (22.3)	0.51 (0.27 to 0.95)	.03
Clinical intrauterine pregnancies	134/443 (30.2)	157/443 (35.4)	0.79 (0.60 to 1.05)	.10
Live births	113/443 (25.5)	115/443 (26.0)	0.98 (0.72 to 1.32)	.88
Worst case for PYG^c^				
Early miscarriages^b^	18/134 (13.4)	36/157 (22.9)	0.52 (0.28 to 0.97)	.04
Clinical intrauterine pregnancies	134/443 (30.2)	157/443 (35.4)	0.79 (0.60 to 1.05)	.10
Live births	112/443 (25.3)	114/443 (25.7)	0.98 (0.72 to 1.32)	.88

PYG, Peiyu Granule.

a The best case for PYG: The 1 unknown event in the PYG group was imputed as live birth and the 1 unknown event in the placebo group was early miscarriage.

b Miscarriage rate was defined as early miscarriages per clinical intrauterine pregnancies.

c The worst case for PYG: The 1 unknown event in the PYG group was imputed as early miscarriage and the 1 unknown event in the placebo group was live birth.

d 95% CIs of ARD were calculated using VassarStats.

## Discussion

*In vitro* fertilization and embryo transfer (IVF-ET) has developed rapidly since its inception 40 years ago. Recently, it has become one of the most commonly used treatments in infertility. However, the success rate of IVF is still 19% to 22% (34, 35). In order to improve success rate, a growing list of adjuvant treatments are used (36, 37). Previous research suggested that prednisone significantly reduced the biochemical pregnancy loss among women with recurrent implantation failure (38).

To our knowledge, this study was the first large-scale, double-blind, placebo-controlled, randomized clinical trial to investigate CHM as a add-on therapy to reduce early miscarriages among women undergoing IVF-ET. Our findings revealed that PYG significantly reduced the early miscarriage rate compared with placebo. 2 participants (1 in intervention group and 1 in control group) lost to follow-up, and we failed to collect their early miscarriage outcome. To minimize the impact of missing data, best-case and worst-case sensitivity analysis were conducted. In best-case analysis, the 1 unknown event in the PYG group was imputed as live birth and the 1 unknown event in the placebo group was early miscarriage. In worst-case analysis, the 1 unknown event in the PYG group was imputed as early miscarriage and the 1 unknown event in the placebo group was live birth. Best-case and worst-case analysis showed similar results to ITT analysis, suggesting stability and incredible of the result.

However, there was no significant between-group difference in clinical pregnancy and live birth. Despite successful randomization, the placebo group exhibited a higher rate of clinical pregnancies (35.2% vs. 30.0%, absolute difference: 5.2%). As the sample size was large enough, the pearson chi-square test was used to compare between-group difference. Given that RR value included one and *P* vaule was greater than 0.05, this difference did not reach statistical significance (RR 0.79, 95% CI 0.60-1.05; p=0.10). Additionally, several factors contributing to this difference should be further considered. PYG group had a higher previous miscarriage rate (47.18% vs. 40.18%, absolute difference: 7%), although no significant difference was observed. Recurrent pregnancy loss can be caused by endometrial dysfunction, which might lead to poor clinical pregnancy outcome (39). Additionally, embryo quality is another potential factor associated with clinical pregnancy (40). Mean grade of transferred embryos has proven to be a well-established, independent predictor of clinical pregnancy rate (40). However, variables such as endometrial receptivity, embryo quality were not detected in baseline assessment. Further exploration of this question is planned in our subsequent studies. Multiple previous studies supported our findings that adjuvant treatments (endometrial scratching, acupuncture, etc.) had no significant effect on clinical pregnancy rate and live birth (33, 37, 41).

Another interesting finding was that PYG reduced early miscarriage but failed to improve clinical pregnancy rates. This might be attributed to the consequence that PYG mainly reduce the risk of early miscarriage by improving the endometrial environment and supporting embryonic stability after implantation. However, as it may not directly influence embryo quality or the implantation process itself, its effect on improving the clinical pregnancy rate remains limited. This is further supported by our previous findings that PYG could improve placental function, enhance the invasion and proliferation of villous trophoblast cells to prevent spontaneous abortion in early pregnancy (29). A retrospective study revealed that wenyang huazhuo compound had significant effect on clinical pregnancy, but no significant effect on early miscarriage in women undergoing ET (42). Another clinical trial demonstrated that self-made ovary-nourishing compound increased high-quality embryo rates, but not early miscarriage rates (43). The differences in therapeutic efficacy may be attributed to the fact that different TCM compounds have distinct mechanisms of action. IVF-ET is a complex and multifaceted procedure that requires targeted TCM compounds at different stages to improve the overall success rate.

Increasing the success rate of IVF-ET is a complicated process that has not yet been thoroughly investigated. Early miscarriage is an important factors affecting the outcome of the procedure (24, 34, 35). It is reported the early miscarriage is negatively associated with live birth (44). Comprehensive basic research has demonstrated multiple regulatory effects of PYG on IVF-ET process. Pharmacological studies show that the flavonoid components contained in Semen Cuscutae have an estrogen-like function, which can improve the reproductive endocrine function (45). The flavonoid glycosides in Herba Taxilli have progesterone-like effects and can supplement the endocrine function of patients (46).

We noticed that more participants refused to take the medicine in PYG group than in placebo group. We re-checked the visual, taste and olfactory indexes of placebo, and all three indexed met our requirements (**Supplementary 3**). Therefore, other contingent factors might result in this difference. Although the dropout rate is low (3.8% vs. 2.3%), related details should be paid more attention to make results more credible and robust.

Although PYG was mainly designed to treat patients with threatened abortion of spleen and kidney deficiency syndrome, its sophisticated formula, which is composed of 19 different herbs, allows them to address a variety of other syndromes. For example, E’jiao and Shanyao possess the function of tonifying Qi and blood; Shudihuang and Heshouwu have the effect of nourishing the liver and kidneys; Qianshi and Sharen are known for their actions in strengthening the spleen and removing dampness. Previous study demonstrated that PYG could enhance invasion and proliferation abilities of human trophoblast cell. Human trophoblast cell plays an important role in early pregnancy (29). Therefore, PYG has its potential effect on conditions beyond spleen-kidney deficiency. Additionally, there is no unified standard for the diagnosis of syndrome types in the population undergoing embryo transfer. RCTs indeed require meticulous design including proper diagnosis. Considering reasons mentioned above, we didn’t add syndrome differentiation into inclusion criteria. However, syndrome differentiation is fundamental principle of TCM practice. Clinical practice guideline or expert consensus for the classification and diagnosis of syndrome differentiation in embryo transfer populations are urgently needed.

Our study has several strengths. First, the IVF-ET protocols are balanced between intervention and control groups, thus excluding potential bias that affected our results Second, sensitivity analysis showed the similar results to the overall findings, indicating the robustness and credibility of each outcome. Third, the trial had a long follow-up duration and period and a high follow-up rate, which allowed for the acquisition of live birth information. Forth, the treatment protocol was formulated by consensus of clinical experts based on optimal practice.

## Limitations

First, this study was a single-center randomized clinical study, and generalization of this finding to other patient populations should be used with caution. However, conducting the study at a single center allowed for greater consistency and control over the quality of laboratory procedures and IVF-ET techniques. To provide more robust evidence for the future clinical application of PYG, a multi-center randomized clinical trial will be necessary. Second, all eligible women who underwent ET received the same TCM formula (PYG, applied to treat patients with threatened abortion of spleen and kidney deficiency syndrome), and we did not take the TCM pattern diagnosis using tools such as pulse, tongue, general physical and emotional wellbeing, etc. However, this is indeed a contradiction that RCTs usually utilize a fixed protocol, while proper diagnosis and individualized protocols are fundamental to the basic principles of TCM. This is a challenge, as some form of uniform IVF plan is required. Third, women in the first trimester had difficulty adhering to medication for the special smell and taste of CHM, sometimes even aggravating the pregnancy reaction. Therefore, future research should focus on how to improve CHM dosage forms to ensure patient adherence to medication. Forth, all patients included in this study received a standardized luteal phase support regimen, which is a commonly used protocol in clinical practice to support early pregnancy (47). While the primary aim of our study was to evaluate the independent effects of PYG on improving reproductive outcomes, the potential interaction between PYG and progesterone warrants further investigation. We recognize that the lack of a control group without progesterone supplementation limited our ability to fully compare the independent effects of PYG versus progesterone, or to assess interactions between the two. This was a limitation of the current study design, primarily due to ethical and clinical considerations, as not providing luteal phase support in women undergoing ET would not align with standard care practices. Fifth, the sample size was calculated based on achieving 175 clinical pregnancies per group. However, actual clinical pregnancies were substantially lower (133 in PYG vs. 156 in placebo), which might influence the reliability of primary outcome. According to the published article (48) and pre-experimental results, we assumed that the early miscarriage was 27.27% among women undergoing ET, and 132 clinical pregnancies per group were required. In our study, there were 133 clinical pregnancies in PYG group and 156 clinical pregnancies in placebo group, suggesting that the sample size is enough for primary outcome. Considering long research period and the development of ET, miscarriage rates of ET might decrease as the trial proceeded. Therefore, we chose a conservative miscarriage rate (22%) for sample size calculation.

## Conclusion

This trial demonstrated that the PYG reduced the early miscarriage rates among women who underwent ET. However, no beneficial effects were observed in clinical pregnancy and live birth. PYG were shown to be effective and safe, offering a promising intervention for women undergoing IVF-ET to improve success rates.

## Data Availability

The raw data supporting the conclusions of this article will be made available by the authors, without undue reservation.
