# Supplementary material for: Effect of the Peiyu granules on early miscarriage among women undergoing embryo transfer: a randomized, double-blind, placebo-controlled trial
Source: Front Endocrinol (Lausanne). 2025 Sep 9;16:1631313. doi: 10.3389/fendo.2025.1631313 (PMC12457714; doi:10.3389/fendo.2025.1631313)
Supplement: Supplementary file 1 [file DataSheet1.pdf]

Effect of Peiyu Granules on Early Miscarriage among Women Undergoing Embryo Transfer: A Randomized, Double-blind, Placebo-controlled Trial

Protocol summary

Objective

To investigate whether progesterone combined with Traditional Chinese Medicine Peiyu granules as luteal phase support improves pregnancy outcomes of women undergoing embryo transfer.

Design

This is a single center, double-blind, parallel-group randomized and controlled trial.

Participants

Beginning from February 2017, all infertile women preparing to receive ET treatment on the Center of Reproductive Medicine of Beijing Obstetrics and Gynecology Hospital, Capital Medical University are candidates for screening. From them total 886 eligible participants will enter this study after careful inquiry for the past history and laboratory assay.

Follow-up would be continued until the end of the last pregnancy. According to our pilot study, up to 440-510 participants could be screened out each year. The duration of recruitment is assumed to be 2 years.

886 eligible women undergoing ET will be randomized to the intervention group and control group. The randomization sequence was computer generated with a computer developed randomization sequence with the ratio of 1:1. In order to ensure allocation concealment, the subject number, random number and corresponding drug number were obtained only by inputting the eligible women's birth date into the Central Randomization System for Clinical Research (Web Edition) developed by the Clinical Evaluation Center of the China Academy of Chinese Medical Sciences.

Inclusion criteria:

1) Aged 22-40 years old;

2) Patients who planned to undergo EF;

3) Body mass index (BMI, calculated as weight in kilograms divided by height in

meters squared) 35 or lower.

Exclusion criteria:

1) Women with 3 or more spontaneous miscarriages;
2) severe endometriosis, including adenomyosis of the uterus and ovarian “chocolate” cysts;
3) anatomical cacogenesis of uterus (eg, uterus unicornis, arcuate uterus, septate uterus, etc);
4) untreated bilateral hydrosalpinx;
5) untreated endometrial diseases (eg, endometritis, endometrial polyp);
6) diseases unsuitable for ART or pregnancy.

Withdrawal criteria

1) Participants are able to voluntarily withdraw from the study at any moment.
2) The researchers consider that some participants should be withdrawn from the study based on medical points of view.
3) Participants who had withdrawn already cannot be reincluded into the study again.

Research procedure

The participants are randomized to either PYG treatment (intervention group, n=443) or the placebo (control group, n=443). PYG or placebo treatment initiates on the ET day with three bags (10.1 g/bag), twice a day. The dosage and method of drugs are exactly same between groups. The first treatment course was from the night of ET to the day of the hCG test (generally 12-14 days after ET). If it was positive (hCG > 10 miu / ml), the second treatment course started and kept the same treatment until the day of transvaginal ultrasound. Medication was continued until the 70th day after ET if women achieved clinical pregnancy. The medication and follow-up ceased when women were not pregnant after ET.

Western medicine luteal support protocol:

Eligible women in both groups received the same western medicine luteal support protocol. Oral progesterone capsules (Zhejiang Xianju Pharmaceutical Co. Ltd. , Chinese medicine approval: H20041902, 100mg each time, twice a day) combined with vaginal progesterone soft capsule (Besins Healthcare Benelux, Imported Drug

Registration No. H20160265, Spain, 0.2g each time, three times a day) from the day of ET until the 10th week of pregnancy.

Dosage

PYG (10.1 g / bag, Jiangyin Tianjiang Medicine Co. Ltd. China), three bags a time, twice a day;

Placebo (10.1 g / bag, Jiangyin Tianjiang Medicine Co. Ltd. China), three bags a time, twice a day;

Oral progesterone capsules (Zhejiang Xianju Pharmaceutical Co. Ltd. Chinese medicine approval: H20041902, 50 mg/grains), 100mg a time, twice a day;

Vaginal progesterone soft capsule (Besins Healthcare Benelux, Imported Drug Registration No. H20160265, Spain, 0.1g/grains), 0.2g a time, three times a day.

Medication dispensing

Following randomization, women with special prescription for this study will be taken to Good Clinical Practice (GCP) pharmacy for PYG or placebo by clinical trial coordinator. The two persons in charge will give the drugs and diary cards to women after checking their basic information and prescription, concurrently informed them of the drugs taking method, matters needing attention and next follow-up time. The remaining drug, packages of the drugs and diary cards which recorded the details of daily medications will be recovered.

Plan of follow-up

The plan of follow-up is listed in **Table 1**.

Table 1.The plan of follow-up

		informed consent	Inclusion criteria	Personal information	History ang physical examination	Drug combination	Pregnancy outcomes	Adverse events
V0	Screening and randomization	√	√	√	√	√	√	
V1	14 ± 2d after ET				√	√	√	√
V2	30 ± 7d after ET				√	√	√	√
V3	70d after ET				√	√	√	√
V4	24 weeks of gestation						√	
V5	Follow-up of the outcomes						√	

Data collection

Demographic characteristics, including age, height, weight, BMI, causes of infertility, pregnancy history and concomitant medications are all collected at the first visit.

Serum HCG and progesterone levels are assayed at the laboratory of Beijing Obstetrics and Gynecology Hospital, Capital Medical University.

All the adverse events need to be recorded.

As to the safety of PYG treatment during pregnancy, there is no clear or consistent evidence of serious adverse effect of PYG on mothers or babies. No fetus toxicity or teratogenicity is reported. However, it cannot be ruled out that there may be some risks, discomfort, drug interactions or adverse reactions that cannot be foreseen at this time, which should be paid attention to and recorded.

All the visits will be recorded in case report forms (CRFs).

Outcomes

Primary outcomes

The primary outcome was early miscarriage, defined as the loss of a viable intrauterine pregnancy up to and including gestational week 12 + 0.

Secondary outcomes

Secondary outcomes included clinical intrauterine pregnancy, late miscarriage and live births. Ultrasonography showing an intrauterine sac with or without a fetal heart could be diagnosed as clinical intrauterine pregnancy [39]. Late miscarriage was defined as pregnancy loss between 13 and 28 weeks of gestation [45]. Live births were defined as the delivery of at least one living infant [46 47]. In addition, women with persistent

pregnancy were followed up until delivery. The delivery mode, gestational age and birth weight were all recorded. The adverse events of the intervention were detailed from the beginning to the end of the study.

Sample size

A survey showed that the risk of pregnancy loss among women under 33 years using their own oocytes and freshly fertilized embryos was 22%[48]. Combined with the pre-experimental results, it was assumed that Peiyu Granules intervention could reduce the risk of pregnancy loss by half. Pregnancy loss rate decreased from 22% to 11%, requiring 175 clinical pregnancies per group (α error, 0.05; β error,0.2). The clinical pregnancy rate at the Center of Reproductive Medicine of Beijing Obstetrics and Gynecology Hospital, Capital Medical University, was 41.5%. And assuming a 5% dropout rate, the total number of women required for randomization was 443 for each group (Table 1.).

The sample size is calculated according the following formula:

$$n = \frac{[u_{\alpha/2}\sqrt{2\bar{p}(1-\bar{p})} + u_{\beta}\sqrt{p_1(1-p_1)+p_2(1-p_2)}]^2}{(p_1-p_2)^2}$$

set α=0.05 (two sided test), $u_{\alpha/2}$=1.96; when β=0.2, u_{β} =0.84; $\bar{p} = \frac{p_1+p_2}{2}$

Table 1. Calculation of sample size

	Miscarriage rate		
α=0.05	Placebo group	Intervention group	Sample size
Events rate	22%	11%	
Power=0.8	175	175	175/0.415×1.05×2=886

Data management

Data collection

The investigators are responsible for recording the complete data of the participants in the CRFs in time. After checked by the monitor, the signed CRFs will be handed to the administrator of data.

Double-entry and double comparison are applied. Any discrepancy needs to be reported to the monitors, and the investigators should answer for it. All questions and replies should be recorded in a query form and saved.

Data verification and management

After all data is confirmed, the administrator should complete a report about the completion of the study (including a drop-off list), check the inclusion/exclusion criteria, integrity, logical consistency, outlier data, time window, accompanied medication and adverse events.

At the audit meeting, the main investigators, monitors, data administrators and statisticians will make resolutions about the informed consents and query forms. After audit meeting, a review of the meeting is documented and the database should be locked.

Data archive

CRFs will be checked and saved in numerical order with search catalogs. Electronic data, including the database, inspection procedure, analysis program, results of analysis, codebook and description files, etc., should be classified and stored. Multiple backups should be saved on different disks or recording media. All original files should be stored within the stipulated time limit.

Data analysis

Intention-to-treat principle (ITT) was applied in our analysis. Data were expressed as mean ± S.D. or n (%). The risk differences (RDs) and relative risks (RRs) with associated 95% CIs were estimated for the primary and secondary outcomes. Comparison between groups was performed with independent sample t test or Mann-Whitney test, or Pearson chi-square test or fisher's exact test as appropriate.

All Statistical analyses were done using the statistical package SPSS, version 26.0 (IBM Corp). Statistical significance was defined as $P < 0.05$ with two tails.

Safety evaluation

PYG is created by a Famous TCM expert Mr. Zhao Songquan of Beijing Obstetrics and Gynecology Hospital, Capital Medical University on the basis of more than 30 years of clinical experience. His treatment experience has been repeatedly verified by many hospitals at home and abroad with remarkable curative effect. After authorization, our hospital made the PYG into in-hospital preparations. Clinical widely used in the treatment of spontaneous abortion, so its possible risk is very small. Pregnancy outcomes are the main outcome measures in our study, and all the adverse

events will be determined if they are correlated with the medication and then documented.

Protection of rights and interests of participants and informed consent

Only after the permission of the ethic committee this study can initiate.

The decisions of the ethics committee, including approval, approval after modifications, disapproval, termination or suspension of the approved trial, must be accepted. It's the responsibility of the involved physicians to provide the patient with details of the clinical trial, including the aims and property of the study, their probable profit and risks, other available therapeutic options, and the rights and obligations of the participants that in accordance with the provisions of the Helsinki declaration, etc. When the participants are fully informed and agree to join in the study, they sign the informed consent and we are allowed to conduct the trial on individuals.

Quality control

The involved physicians should be trained before the trial and remain relatively fixed. The participants are requested to have a visit on time as programmed. For patients who do not visit on time, the investigators should follow up. It's the responsibility of the investigators to fully inform the patients and their relatives the meaning of the trial, and to promote the compliance of the patients and insure medication taking on time.

Premature termination of the study

The trial must be terminated in time if serious safety problem presented.

The trial must be terminated if the intervention shows very poor treatment effect, or even no effect.

The trial must be terminated if major errors are presented in the clinical trial, or serious deviations are found in the implementation of the trial resulting in a difficulty in evaluating the effect of the medication.
