# Supplementary material for: Effect of the Peiyu granules on early miscarriage among women undergoing embryo transfer: a randomized, double-blind, placebo-controlled trial
Source: Front Endocrinol (Lausanne). 2025 Sep 9;16:1631313. doi: 10.3389/fendo.2025.1631313 (PMC12457714; doi:10.3389/fendo.2025.1631313)
Supplement: Supplementary file 2 [file DataSheet2.pdf]

Supplementary materials

Table S1. The composition of PYG [a]

Component			
**Latin name**	**Species**	**Family**	**Ratio (%) [b,c]**
Herba Taxilli	Taxillus chinensis (DC.) Danser	Loranthaceae	6.1
Semen Cuscutae	Cuscuta chinensis Lam.	Convolvulaceae	5.1
Cortex Eucommiae	Eucommia ulmoides Oliv.	Eucommiaceae	5.1
Radix Dipsaci	Dipsacus asper Wall.ex Henry	Dipsacaceae	5.1
Rhizoma Dioscoreae	Dioscorea opposita Thunb.	Dioscoreaceae	7.7
Rhizoma Polygonati	Polygonatum sibiricum Red.	Liliaceae	5.1
Semen Eutyales	Euryale ferox Salisb.	Nymphaeaceae	7.7
Radix boehmeriae	Boehmeria nivea （L.） Gaud.	Urticaceae	5.1
Cortex Ailanthi	Ailanthus altissima (Mill.) Swingle	Simarubaceae	5.1
Rhizoma Cimicifugae	Cimicifuga foetida L.	Ranunculaceae	3.1
Radix Rehmanniae	Rehmannia glatinosa Libosch.	Scrophulariaceae	5.1
Radix Rehmanniae preparata	Rehmannia glatinosa Libosch.	Scrophulariaceae	5.1
Fructrs Amomi	Amomum villosum Lour.	Zingiberaceae	1.5
Fructus Corni	Cornus officinalis Sieb. et Zucc.	Cornaceae	5.1
Radix Polygoni Multiflori	Polygonum multiflorum Thunb.	Polygonaceae	5.6
Colla Corii Asini	Equus asinus L.	Equidae	7.1
Caulis Perillae	Perilla frutescens (L.) Britt.	Labiatae	2.6
Receptaculum Nelumbinis	Nelumbo nucifera Gaertn.	Nymphaeaceae	7.7
Radix Pseudostellariae	Pseudostellaria heterophylla (Miq.) Pax	Caryophyllaceae	5.1

[a] Abbreviations: PYG, Peiyu Granule.

[b] Ratio (%) is equaled to the weight of a single herb accounting for the total weight of a dose.

[c] Percentages may not total 100 because of rounding.

TCM - PYG formula preparations

In TCM, the decoction is prepared by boiling in water for hours. However, PYG in this study is a granular formulation manufactured by Jiangyin Tianjiang Medicine Co. Ltd. (China). PYG is made up of 19 kinds of herbs and the ratio is presented in Table S1. It is used in accordance with the processing standards of Chinese herbal piece as raw materials, and refined by modern pharmaceutical technologies, such as extraction, concentration, drying, and packaging. It ensures all the characteristics of the original Chinese herbal pieces and does not need to be decocted and taken directly. It has the

advantages of a smaller dosage, rapid action, and convenient carrying and preservation. It is more suitable for modern people to use.

Placebo preparations

The placebo was also provided by Jiangyin Tianjiang Medicine Co. Ltd. (China). The placebo was a mixture of 50% starch, 40% caramel and 10% of the active PYG ingredients mixed, dried, crushed, and lumped together. Patients in the placebo group consumed the same amount of placebo as the treatment group. The placebo and the herbal medicines used to make PYG are identical in appearance, colour, smell, taste, packaging, usage and dosage.

Table S2. Baseline characteristics of the participants in intervention and control groups (Per-protocol analysis)

Characteristic	Intervention (n = 423)	Control (n = 431)
Age, mean (SD), y	32.8 (3.6)	32.8 (3.6)
Body mass index, mean (SD) [a]	23.3 (3.8)	23.3 (3.9)
Primary infertility, No. (%)	209 (49.4)	233 (54.1)
Duration of infertility, median (IQR), y	3 (2-5)	4 (2-5)
Previous spontaneous miscarriages, No. (%)		
0	223 (52.7)	256 (59.4)
1	126 (29.8)	105 (24.4)
2	64 (15.1)	62 (14.4)
3	10 (2.4)	8 (1.9)
Occupation, No. (%)		
Unemployed	123 (29.1)	124 (28.8)
Blue-collar worker	41 (9.7)	33 (7.7)
White-collar worker	259 (61.2)	274 (63.6)
Education background, No. (%)		
Junior high school and below	41 (9.7)	44 (10.2)
Senior high school	92 (21.7)	85 (19.7)
University degree or above	290 (68.6)	302 (70.1)
Causes of infertility, No. (%)		
Male factors	159 (21.9)	177 (24.2)
Female factors		
Tubal factor	341 (47.0)	314 (42.9)
Endometriosis	26 (3.6)	32 (4.4)
Diminished ovarian reserve	45 (6.2)	37 (5.1)
Polycystic ovary syndrome	57 (7.9)	70 (9.6)
Intrauterine insemination failure	77 (10.6)	80 (10.9)
Unknown factors	20 (2.8)	22 (3.0)
Follicle-stimulating hormone, median (IQR), IU/L	6.99 (5.92-7.98)	6.97 (5.85-8.32)
Luteinizing hormone, median (IQR), IU/L	3.28 (2.81-5.25)	3.88 (2.82-5.29)
Estradiol, median (IQR), pg/mL	43.51 (35.65-52.69)	42.95 (34.21-53.43)
Testosterone, median (IQR), ng/dL	36.86 (28.00-46.57)	36.57 (28.86-46.00)
Prolactin, median (IQR), ng/mL	10.93 (8.28-15.31)	11.55 (8.58-16.63)
Progesterone, median (IQR), ng/mL	0.53 (0.38-0.71)	0.50 (0.37-0.71)
Thyroid-stimulating hormone, median (IQR), mIU/L	1.94 (1.45-2.60)	1.95 (1.44-2.69)

Abbreviation: SD, standard deviation; IQR, interquartile range.

[a] Body mass index is the weight in kilograms divided by the square of the height in meters.

Table S3. Protocols of Controlled Ovarian Hyperstimulation and Data of In Vitro Fertilization and Embryo Transfer (Per-protocol analysis)

Characteristic	No. (%) of Participants		*P* value
	Intervention (n = 423)	Control (n = 431)	
Types of embryo transfer			
Fresh embryo transfer	152 (35.9)	164 (38.1)	.52
Frozen thawed embryo transfer	271 (64.1)	267 (61.9)	
Hormone replacement of frozen thawed embryo transfer	166 (61.3)	170 (63.7)	.56
Natural cycle of frozen thawed embryo transfer	105 (38.7)	97 (36.3)	
Protocol of controlled ovarian hyperstimulation			
Ultra-long GnRH agonist	40 (9.5)	50 (11.6)	.78
Long GnRH agonist	131 (31.0)	127 (29.5)	
Short GnRH agonist	54 (12.8)	54 (12.5)	
GnRH antagonist	198 (46.8)	200 (46.4)	
Times of ET			
0	190 (44.9)	204 (47.3)	.72
1	123 (29.1)	115 (26.7)	
2	62 (14.7)	69 (16.0)	
3 or more	548(11.3)	43 (10.0)	
Number of embryos transferred			
1	90 (21.3)	85 (19.7)	.52 [a]
2	332 (78.5)	343 (79.6)	
3	1 (0.2)	3 (0.7)	
Stage of embryos transferred			
Blastocyst stage	343 (81.1)	335 (77.7)	.22
Cleavage stage	80 (18.9)	96 (22.3)	

Abbreviation: GnRH, gonadotropin-releasing hormone; ET, embryo transfer.

[a] Fisher exact test.

Table S4. Pregnancy Outcomes of the Participants (Per-protocol analysis)

IVF-ET outcomes	No. / Total No. (%) Intervention	No. / Total No. (%) Control	Relative Risk (RR), (95% CI)	*P* Value
Primary outcome				
Miscarriages [a]	19/130 (14.6)	38/150 (25.3)	0.50 (0.27 to 0.93)	.03
Early	16/130 (12.3)	33/150 (22.0)	0.50 (0.26 to 0.95)	.03
Late	3/130 (2.3)	5/150 (3.3)	0.69 (0.16 to 2.92)	.73 [e]
Secondary Outcome				
Clinical intrauterine pregnancies	130/423 (30.7)	150/431 (34.8)	0.83 (0.62 to 1.11)	.21
Twin pregnancies [b]	26/130 (20.0)	32/150 (21.3)	0.92 (0.52 to 1.65)	.78
Live births	110/423 (26.0)	111/431 (25.8)	1.01 (0.75 to 1.38)	.93
Preterm deliveries [c]	33/110 (30.0)	37/111 (33.3)	0.86 (0.49 to 1.51)	.59
Birth weight (g)				
Singleton	3,350 (3,053 - 3,588)	3,410 (3,150 - 3,753)		.35
Twin	2,623 (2,284 - 2,900)	2,515 (2,275 - 2,699)		.19
Adverse event				
Gastrointestinal discomfort [f]	71/423 (16.8)	93/431 (21.6)	0.78 (0.59 to 1.03)	.08

P＜ .05 was considered statistically significant.

[a] Miscarriage was defined as pregnancy loss before the 28th week of gestation; the miscarriage rate was defined as miscarriages per clinical intrauterine pregnancy.

[b] Twin pregnancy rate was defined as twin pregnancies per clinical intrauterine pregnancies.

[c] Premature delivery was defined as a live birth before 37 weeks of gestation; Preterm delivery rate was defined as preterm deliveries per live births.

[d] 95% CIs of ARD were calculated using VassarStats

[e] Fisher exact test

[f] Gastrointestinal discomfort is mainly manifested as diarrhea with or without abdominal distension, nausea.

Table S5. Post Hoc Sensitivity Analysis for Pregnancy Outcomes of the Participants (Intention-to-Treat Analysis)

IVF-ET outcomes	No. / Total No. (%) Intervention	Control	Relative Risk (RR), (95% CI)	*P* Value
Best case for PYG [a]				
Early miscarriages [b]	17/134 (7.9)	36/157 (17.4)	0.49 (0.26 to 0.92)	.02
Clinical intrauterine pregnancies	134/443 (30.2)	157/443 (35.4)	0.79 (0.60 to 1.05)	.10
Live births	113/443 (25.5)	114/443 (25.7)	0.99 (0.73 to 1.34)	.94
Worst case for PYG [c]				
Early miscarriages [b]	18/134 (13.4)	35/157 (22.3)	0.54 (0.29 to 1.01)	.05
Clinical intrauterine pregnancies	134/443 (30.2)	157/443 (35.4)	0.79 (0.60 to 1.05)	.10
Live births	112/443 (25.3)	115/443 (26.0)	0.97 (0.71 to 1.30)	.82

Abbreviation: PYG, Peiyu Granule.

[a] The best case for PYG: The 1 unknown event in the PYG group was imputed as live birth and the 1 unknown event in the placebo group was early miscarriage.

[b] Miscarriage rate was defined as early miscarriages per clinical intrauterine pregnancies.

[c] The worst case for PYG: The 1 unknown event in the PYG group was imputed as early miscarriage and the 1 unknown event in the placebo group was live birth.

[d] 95% CIs of ARD were calculated using VassarStats

Table S6. Post Hoc Sensitivity Analysis for Pregnancy Outcomes of the Participants (Per-protocol analysis)

	No. / Total No. (%)			
**IVF-ET outcomes**	**Intervention**	**Control**	**Relative Risk (RR), (95% CI)**	***P* Value**
Best case for PYG [a]				
Early miscarriages [b]	16/131 (12.2)	34 /151 (22.5)	0.48 (0.25 to 0.91)	.02
Clinical intrauterine pregnancies	131/424 (30.9)	151/432 (35.0)	0.83 (0.63 to 1.11)	.21
Live births	111/424 (26.2)	111/432 (25.7)	1.03 (0.76 to 1.39)	.87
Worst case for PYG [c]				
Early miscarriages [b]	17/131 (13.0)	33/151 (21.9)	0.53 (0.28 to 1.01)	.05
Clinical intrauterine pregnancies	131/424 (30.9)	151/432 (35.0)	0.83 (0.63 to 1.11)	.21
Live births	111/424 (25.9)	113/432 (25.9)	1.00 (0.74 to 1.36)	1.00

Abbreviation: PYG, Peiyu Granule.

[a] The best case for PYG: The 1 unknown event in the PYG group was imputed as live birth and the 1 unknown event in the placebo group was early miscarriage.

[b] Miscarriage rate was defined as early miscarriages per clinical intrauterine pregnancies.

[c] The worst case for PYG: The 1 unknown event in the PYG group was imputed as early miscarriage and the 1 unknown event in the placebo group was live birth.

[d] 95% CIs of ARD were calculated using VassarStats

Table S7. Adverse Events

	PYG group (n=443)	Placebo group (n=443)
Any adverse events	71/443 (16.0)	93/443 (21.0)
Serious adverse events	0/443 (0.0)	0/443 (0.0)
Fatal events	0/443 (0.0)	0/443 (0.0)
Adverse events reported in patients		
Diarrhea	52/443 (11.7)	81/443 (18.3)
Stomach discomfort	9/443 (2.0)	6/443 (1.4)
Haemorrhagia	5/443 (1.1)	4/443 (0.9)
Loose stool	5/443 (1.1)	3/443 (0.7)
Abdominal distension	6/443 (1.4)	2/443 (0.5)
Bellyache	5/443 1.1)	2/443 (0.5)
Stomachach	1/443 (0.0)	2/443 (0.5)
Constipation	2/443 (0.5)	1/443 (0.2)
Vomit	1/443 (0.2)	2/443 (0.5)
Borborygmus	0/443 (0.0)	2/443 (0.5)
Nausea	1/443 (0.2)	0/443 (0.0)
Thirst	0/443 (0.0)	1/443 (0.2)
Belching	1/443 (0.2)	0/443 (0.0)
Farting	0/443 (0.0)	1/443 (0.2)
Halitosis	0/443 (0.0)	1/443 (0.2)
Dryness-heat	1/443 (0.2)	0/443 (0.0)
Safety areas of interest		
Gastrointestinal disorders	71/443 (16.0)	93/443 (21.0)
Hematologic disorders	5/443 (1.1)	4/443 (0.9)

Abbreviation: PYG, Peiyu Granule.

Data are n (%) of the safety analysis population (all randomly allocated participants exposed to at least one dose of intervention) experiencing at least one event.

Data are for on-treatment adverse events occurring during treatment.

All adverse events were confirmed by event adjudication committee.
