# Supplementary material for: Effect of the Peiyu granules on early miscarriage among women undergoing embryo transfer: a randomized, double-blind, placebo-controlled trial
Source: Front Endocrinol (Lausanne). 2025 Sep 9;16:1631313. doi: 10.3389/fendo.2025.1631313 (PMC12457714; doi:10.3389/fendo.2025.1631313)
Supplement: Supplementary file 3 [file DataSheet3.docx]

**Information of Placebo**

**1. Name and amount of each ingredient**

Starch: 50%

Caramel: 40%

Active PYG ingredients: 10%

**2. Description of the similarity of placebo with PYG**

After comparison, the placebo and PYG are consistent in terms of ppearance, colour, smell, taste, packaging, usage and dosage.

**3. Quality control and safety assessment, if any**

No.

**4. Administration route, regimen, and dosage**

Take the medicine orally after mixing it with hot water, a dose of 3 bags (10.1 g/bag), 2 times a day.

**5. Production information: where, when, how, and by whom the placebo was produced**

**Production date**: September 10, 2016; September 11, 2016

**Manufacturer**: Jiangyin Tianjiang Medicine Co. Ltd.

**Address**: No. 1, Xinsheng Road, High-tech Development Zone, Jiangyin City, Jiangsu Province, China

**6. Components of Jinlida granules Placebo and Evaluation Methods**

**Evaluation Methods:**

1）The visual, taste, and olfactory indexes of placebo and PYG investigational drug are measured independently by visual, taste and olfactory sensors and 10 people’s experiences, and the similarity between them is calculated. For various indexes, the overall scores, objective and quantified scores and human experience sensory scores are given; when the average scores are all greater than 80%, the products are qualified.

2）The weights of visual, taste and olfactory indexes are 40%, 40%, and 20%, respectively.

3）A total of five groups (groups A, B, C, D and E) of placebos are prepared, and compared with PYG investigational drugs.

**Evaluation Results:**Independent assessment is performed based on the above criteria. The objective, quantitative similarities are as follows: A: 92%, C: 85%, D: 81%, E: 75%, B: 69%.

The average sensory scores based on human experiences are

as follows: A: 90%, C: 82%, D: 80%, E: 73%, B: 61%.

**Note:** If none of the five groups meet the conditions, the manufacturing process of placebo will be adjusted, and

placebos will be remanufactured until compliance with requirements.

**Summary:** Through the above evaluation, the placebo A is used and the placebo C is for standby in this study
